# Estimating Typical Multiple Sclerosis Disability Progression Speed from Clinical Observations

**DOI:** 10.1371/journal.pone.0105123

**Published:** 2014-10-17

**Authors:** Murray G. Brown, Mark Asbridge, Vern Hicks, Sarah Kirby, Thomas J. Murray, Pantelis Andreou, Dong Lin

**Affiliations:** 1 Department of Community Health and Epidemiology, Dalhousie University and Capital District Health Authority, Centre for Clinical Research, Halifax, Nova Scotia, Canada; 2 Department of Community Health and Epidemiology and Department of Emergency Medicine, Dalhousie University, Halifax, Nova Scotia, Canada; 3 Department of Community Health and Epidemiology, Dalhousie University, Halifax, Nova Scotia, Canada; 4 Division of Neurology, Dalhousie University, Halifax, Nova Scotia, Canada; 5 Division of Neurology and Department of Community Health and Epidemiology, Dalhousie University, Halifax, Nova Scotia, Canada; 6 Department of Mathematics and Statistics graduate student, Dalhousie University, Halifax, Nova Scotia, Canada; Cardiff University, United Kingdom

## Abstract

**Introduction:**

Multiple sclerosis (MS) is a chronic disease of the central nervous system. Estimates of MS natural history (NH) disability progression speed from clinical observations vary worldwide. This may reflect, in part, variance in censoring-bias) (missing observations) and assumptions about when irreversible disability progression events occurred. We test whether estimates of progression speed which assume midpoint survival time at irreversible disability endpoints are significantly faster than estimates which assume maximum survival time, and are more stable across study groups and time periods.

**Methods:**

Our Nova Scotia NH study population includes 2,240 definite relapsing-onset multiple sclerosis (R-MS) natural history patients with 18,078 Expanded Disability Status Scale (EDSS) clinical observations in study period 1979–2010. Progression speed is measured by rate-of-change in range EDSS 0–6 and by survival time at irreversible endpoints EDSS 1–9. Midpoint censoring-bias-reduction methods are applied to clinical observations.

**Findings:**

Typical EDSS increase per year in range EDSS 0–6, assuming midpoint survival time, is estimated to be 0.168 for all R-MS, 0.204 for eventually-DMD-treated patients and 0.155 for never-DMD-treated patients. Estimates assuming midpoint rather than maximum survival time are significantly faster: 16% faster for all R-MS natural history patients, 6% faster for eventually-DMD-treated patients, and 21% faster for never-DMD-treated patients. The variability of estimates across study groups and time periods decreased when midpoint survival time was assumed.

**Conclusions:**

Estimates of typical disease progression speed from 1979–2010 Nova Scotia clinical observations are sensitive to censoring-bias and to analysts’ survival time assumptions. Censoring-bias-adjusted estimates of typical natural history disability progression speed in relapsing-onset multiple sclerosis patients are significantly faster, and less variable within and across study groups and time periods, than unadjusted estimates, and are, arguably, more relevant for various stakeholders. The application of censoring-bias-reduction methods to other multiple sclerosis clinical databases may reduce variability in estimates of disability progression speed worldwide.

## Introduction

Estimating the typical progression speed of a disease from intermittent clinical observations is inherently uncertain. The exact times of events of interest are not known, but are known to lie in the intervals between certain observations. Consequently, when modeling progression speed from clinical observations, analysts are forced to make assumptions about when irreversible progression events occurred during an interval. They can assume maximum survival time, minimum survival time, or somewhere in between [1].

Estimates of disease progression speed from unadjusted clinical observations implicitly assume maximum survival time – that each patient’s disease does not progress (as measured at their previous clinic visit) until immediately before their next clinic visit. In fact, only rarely do patients experience maximum survival times. In reality, their disease may progress at any point during the interval. Assuming maximal survival times will underestimate disease progression speed. The longer the interval is between observations, the larger the interval censoring bias and the larger the underestimation. Missing (censored) information between experimental study observations collected prospectively at frequent and regular intervals over short study periods is typically small, by design.

Assuming midpoint survival time at events of interest, rather than maximum survival time, is a basic censoring-bias-reduction strategy. If censoring-bias is considered negligible, for study purposes, analysts may choose to accept observations at face value, implicitly assuming maximum survival time. If censoring-bias is considered too large to ignore, analysts may apply censoring-bias-reduction methods which assume that typical survival time lies at the midpoint between minimum and maximum survival time. Post-hoc approaches for addressing censoring-bias in data are not new, and methods which reduce such bias have existed for many years [2]–[3]; yet, the application of these methods in health research remains somewhat limited. Health research papers employing methods to reduce censoring-bias have examined respiratory conditions [4], HIV infection [5], injury risk [6], environment health conditions [7]–[8], substance use [9], and cancer epidemiology [10]. To our knowledge this is the first study to apply censoring-bias reduction methods to multiple sclerosis clinical observations.

Multiple sclerosis (MS) is a chronic disease of the central nervous system. Severe disability develops in most patients within 20 years of onset [11]–[12]. Estimates of natural history disability progression speed from clinical observations vary greatly, within and across clinical databases worldwide [13]–[34]. This variability may reflect, in part, both variance in the frequency and regularity of clinic visits within and across clinical databases and time periods and analysts’ assumption of maximum survival time. Assuming maximum survival time gives downward-biased estimates of typical progression speed. This downward bias varies with censoring-bias-size, which confounds the comparability of progression speed estimates across study groups, time periods and clinical databases.

We test whether estimates of progression speed which assume midpoint survival time are significantly faster – and more stable across study groups and time periods – than estimates which assume maximum survival time. Clinical observations collected at 1979–2010 Nova Scotia’s Dalhousie Multiple Sclerosis Research Unit (DMSRU) clinics are analyzed. The frequency and regularity of visits changed over time as the DMSRU evolved from a solo neurological referral practice, established in 1979, to a large university-hospital-based referral practice serving over 90% of definite MS patients in Nova Scotia in 2010. We discuss the implications of Nova Scotia results for observational studies of disease progression speed and the effectiveness of treatments designed to slow progression speed.

## Methods

### Measuring disability in multiple sclerosis

Neurological disability in multiple sclerosis is measured by the Disability Status Scale (DSS) or Expanded Disability Status Scale (EDSS) [35]–[37], used worldwide for sixty years [38]–[39]. The EDSS is an ordinal scale from 0 (no neurological disability) to10 (death due to MS) with half-point intervals from 1–10. It has near-linear (equal-interval) measurement properties in range EDSS 0–6.5, giving empirical validity to arithmetic operations in this range [40] (Table S1).

### Natural history study population

**Figure 1:**
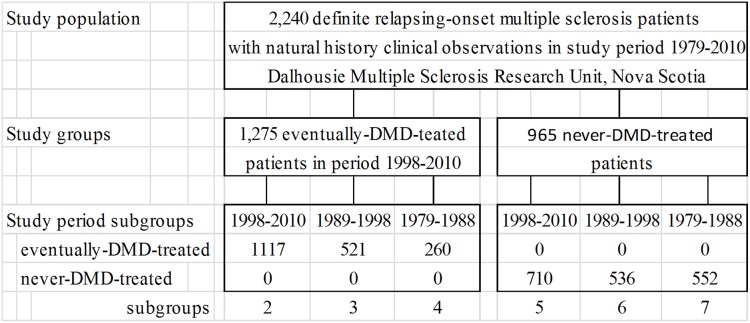
Natural history study population, study groups and study periods. Footnote: DMD = disease-modifying-drug(s). EDSS = Expanded disability Status Scale. The study population includes 2,240 definite relapsing-onset (R-MS) patients who attended DMSRU clinics in study period 1979–31 December2010 while receiving regular care, but not DMDs. This natural history population includes 29 eventually-DMD-treated R-MS patients who were later reclassified as primary progressive. 1,275 R-MS patients were eventually-treated with DMDs under Nova Scotia’s Special Therapies Program in period 1 Aug1998 to 31 Dec2010. 965 R-MS patients were never-treated with DMDs. Unique patients may be included in more than one study groups, depending on when their observations were collected. For example, a patient with at least one EDSS observation in study period 1979–1988 may or may not have observations in period 1989–1998 or 1998–2010.

The study population includes 2,240 unique definite relapsing-onset multiple sclerosis [41]–[43] natural history patients who ever attended referral clinics of the Dalhousie Multiple Sclerosis Research Unit (DMSRU), Nova Scotia, Canada, in study period 1979–2010. (Figure 1) Natural history patients are defined to be patients receiving regular care, but not disease-modifying-drugs (DMDs), when their EDSS was assessed. EDSS was assessed at each clinic visit. On 1 August 1998, Nova Scotia’s Special Therapies Program provided publicly-funded universal-access zero-copayment insurance coverage for first-line disease-modifying-drugs (DMDs) – interferon β1a, interferon β1b, and glatiramer acetate – designed to slow progression in relapsing-onset multiple sclerosis. This program was delivered exclusively through DMSRU clinics.

### Study groups and study periods

Disability progression speed, as measured by EDSS annual change in range EDSS 0–6, is estimated for all 2,240 R-MS patients in our natural history study population, for 1,275 patients eventually-treated with DMDs in period 1998–2010, and for 965 patients never-treated with DMDs. Disability progression speed, as measured by survival time at irreversible EDSS endpoints, is estimated for a synthetic onset cohort of 408 R-MS natural history patients with at least three clinical observations, using Kaplan-Meier methods.

When reporting EDSS assessment rates, ever-DMD-treated and never-DMD-treated study groups are divided into 1998–2010, 1989–1998 and 1979–1988 subgroups, depending upon when at least some of their EDSS observations were collected. Unique patients may be included in more than one subgroup.

### Survival time

Failure to survive at an irreversible disability endpoint (an irreversible progression event) occurs when a patient progresses to a higher endpoint and never reverts to a lower endpoint. The probability of surviving an irreversible endpoint (p) is p = 1–f, where f is the probability of failure to survive.

### Irreversible progression paths – assuming maximum, minimum or midpoint survival time

**Figure 2:**
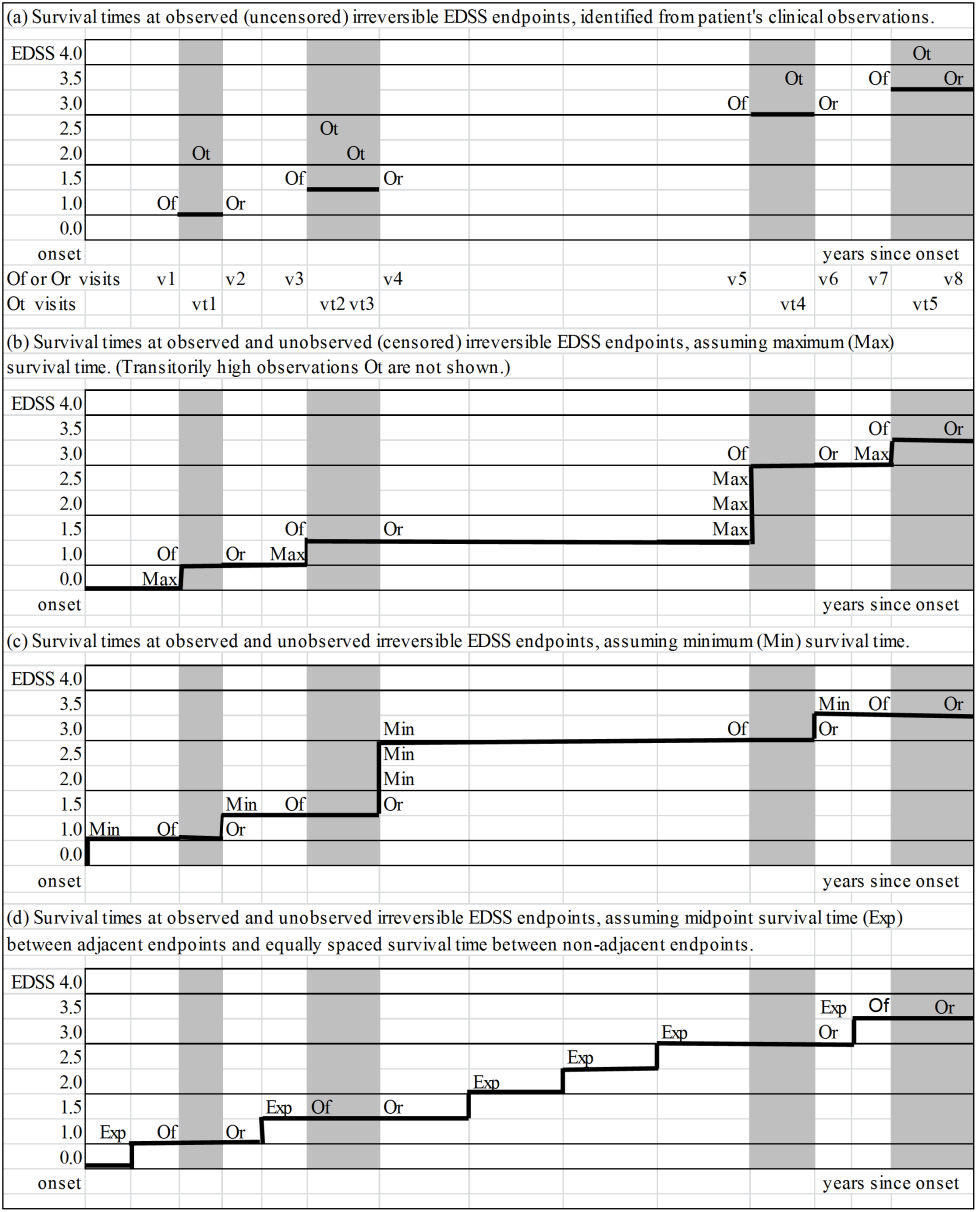
Irreversible progression paths – assuming maximum, minimum or midpoint survival time. Footnote: Part (a) shows a hypothetical multiple sclerosis patient’s clinical observations by years since onset. EDSS = Expanded Disability Status Scale. EDSS = 0 at onset is assumed. Of = first clinical observation at an irreversible EDSS endpoint. Or = last repeat clinical observation at an irreversible EDSS endpoint (repeat clinical observations between Of and Or are not shown). Ot = a transitorily high EDSS clinical observation (Ot are not shown in parts (b), (c) and (d)). Max = maximum survival time. Min = minimum survival time. Exp = expected midpoint survival time. **bold lines** = irreversible disability progression paths.

A hypothetical patient’s clinical observations and survival times at observed irreversible EDSS endpoints are illustrated by **bold lines** in Figure 2, Part (a). Part (b) shows the patient’s irreversible progression path and survival times at observed and unobserved (censored) irreversible EDSS endpoints, assuming maximum survival time. Irreversible disability severity is modeled to remain as measured at a previous visit until the day of the next visit. This gives a slowest-possible estimate of progression speed. Part (c) shows the patient’s progression path when minimum survival time is assumed. Irreversible disability progression is modeled to occur on the day of a clinic visit, but after the visit. This gives a fastest-possible estimate of progression speed. Part (d) shows the patient’s progression path when midpoint survival time is assumed. Irreversible progression events are modeled to occur midway between adjacent observed irreversible endpoints (e.g. 1.0 and 1.5) and at equal intervals between non-adjacent irreversible endpoints (e.g. 1.5 and 3.0) Assuming midpoint survival time gives an estimate of typical progression speed, when actual times of progression events are unknown.

Censoring-bias-adjusted estimates of progression speed which assume midpoint survival time are necessarily faster than estimates which assume maximum survival time, but they are not necessarily significantly faster – from a statistical, clinical or evaluation perspective. We created expected midpoint survival time measures for each patient in the Nova Scotia study population from available 1979–2010 clinical observations. The SAS code which does this is available from the Corresponding Author [44]–[45]. Figure S1 and Figure S2 show irreversible disability progression paths from MS symptom onset, assuming midpoint survival time, for four definite multiple sclerosis patients who attended Dalhousie Multiple Sclerosis Research Unit referral-clinics in period 1979–2010,.

### Progression speed estimation models

Progression speed, as measured by EDSS annual change in range EDSS 0–6, is estimated using a fixed effects parametric regression model. Progression speed, as measured by survival time at irreversible EDSS endpoints, is estimated using a Kaplan-Meier non-parametric model [46].

### Populating estimation models

Estimation models that assume maximum survival time are populated with clinical observations only. Estimation models that assume midpoint survival time are populated with both clinical observations and expected midpoint survival time measures.

### Other types of bias

Estimates of disease progression speed from clinical observations are subject to various types of sampling bias, besides interval-censoring bias. These include demographic bias, immigration bias, ascertainment bias, referral bias, immortal time bias, and lost to follow up. Our study focuses on interval-censoring-bias adjustment methods, holding other sources of bias constant.

### Study approval

Access to Dalhousie Multiple Sclerosis Research Unit (DMSRU) clinical records was approved after submitting a research proposal to the Research Ethics Board, Capital District Health Authority, Department of Health and Wellness, Nova Scotia. These clinical records are the property of the Department of Health and Wellness. The DMSRU obtained written consent from patients (using consent forms and protocols approved by the Research Ethics Board) whenever possible (or from next of kin or caregiver in the case of children) for clinical records to be used in approved research studies. The clinical records of patients who did not consent were excluded from our study population. Clinical records were anonomized before being made available.

## Results

### Rate-of-change estimates

**Figure 3:**
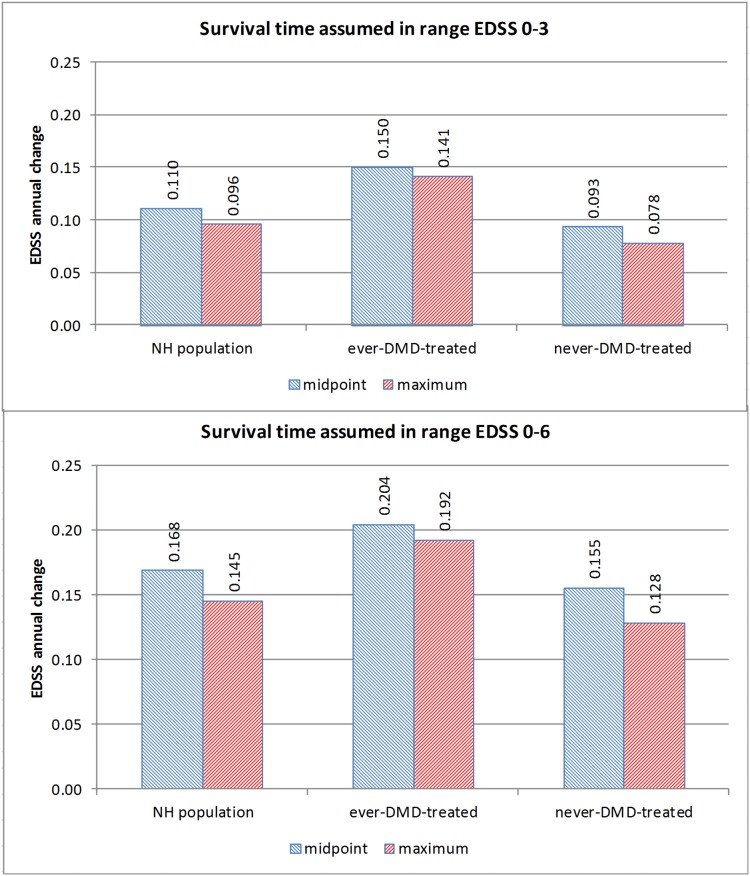
Fixed effects model estimates of disability progression per year in relapsing-onset MS natural history patients, by study groups and time periods, assuming midpoint or maximum survival time, Nova Scotia, 1979–2010. Footnote: EDSS = Expanded Disability Status Scale.

Typical EDSS increase per year in range EDSS 0–6, assuming midpoint survival time at irreversible disability endpoints, is estimated to be 0.168 for all definite relapsing-onset MS natural history patients, 0.204 for eventually-DMD-treated patients and 0.155 for never-DMD-treated patients. Estimates assuming midpoint survival time rather than maximum survival time are significantly faster: 16% faster for all 2,240 R-MS natural history patients, 6% faster for 1,275 eventually-DMD-treated patients, and 21% faster for 965 never-DMD-treated patients. The variability in progression speed estimates across study groups and time periods decreased when midpoint survival time was assumed (Figure 3 and Table S2).

### Survival time estimates

**Figure 4:**
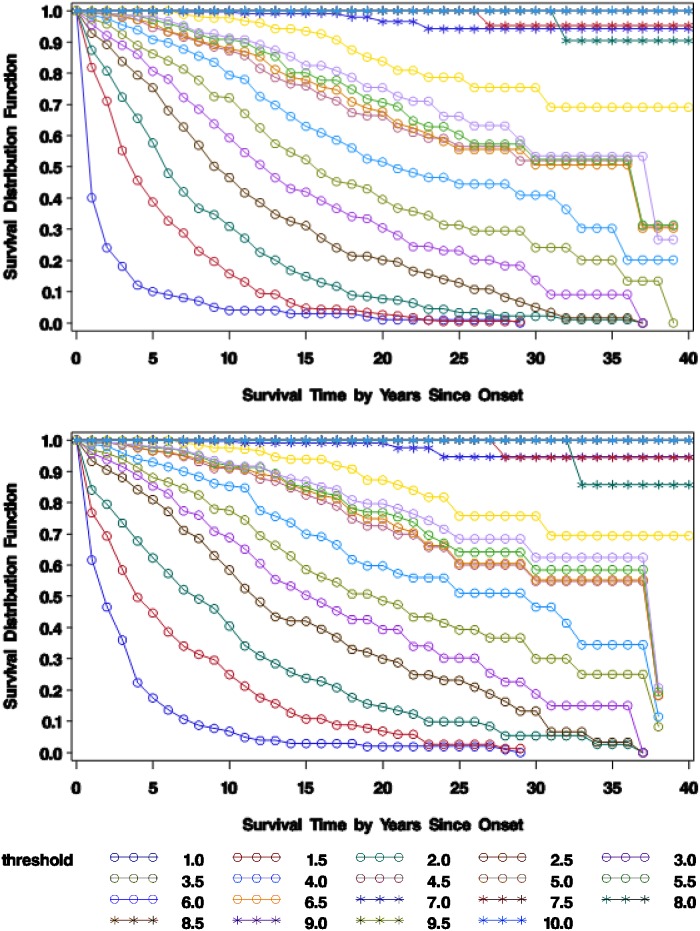
Kaplan-Meier estimates of natural history survival time at EDSS disability endpoints for 408 eventually-DMD-treated patients, assuming midpoint (top) or maximum (bottom) survival time, Nova Scotia, 1979–2010. Footnote: The study group is a synthetic onset cohort of 408 definite relapsing-onset multiple sclerosis patients who were eventually treated with disease-modifying-drugs in period 1 August 1998–31 December 2010 and who had an assessed year-of-onset and at least two natural history EDSS clinical observations in period 1979–2010. The top graph shows Kaplan-Meier survival distribution function estimates at irreversible endpoints EDSS 1–9, assuming midpoint survival time (obs+Exp = 3702). The bottom graph shows Kaplan-Meier survival distribution function estimates at irreversible endpoints EDSS 1–9, assuming maximum survival time (obs = 2170). obs = an EDSS clinical observation, including assessed year-of-onset. Exp = an expected EDSS midpoint measure.

Kaplan-Meier estimates of survival time at irreversible disability endpoints are shorter when midpoint survival time is assumed rather than maximum survival time. Figure 4 shows estimates of natural history survival times at endpoints EDSS 1–9, from 1979–2010 observations, for a synthetic onset cohort of 408 R-MS patients who were eventually-treated with disease-modifying-drugs in period 8/1998–2010, assuming midpoint or maximum survival time. For each disability endpoint, the estimated midpoint-survival- curve lies to the left of the maximumsurvivalcurve, signifying faster progression. The differences between midpoint and maximum survival curve estimates are significantly larger in disability range EDSS 0–3 than in disability range EDSS 3.5–6, and become progressively smaller beyond EDSS 6; this pattern may reflect, in part, the Kaplan-Meier assumption of maximum survival time at right-censored irreversible endpoints, which is not overridden by our interval-censoring bias-reduction methods.

### Censoring-bias measures

**Figure 5:**
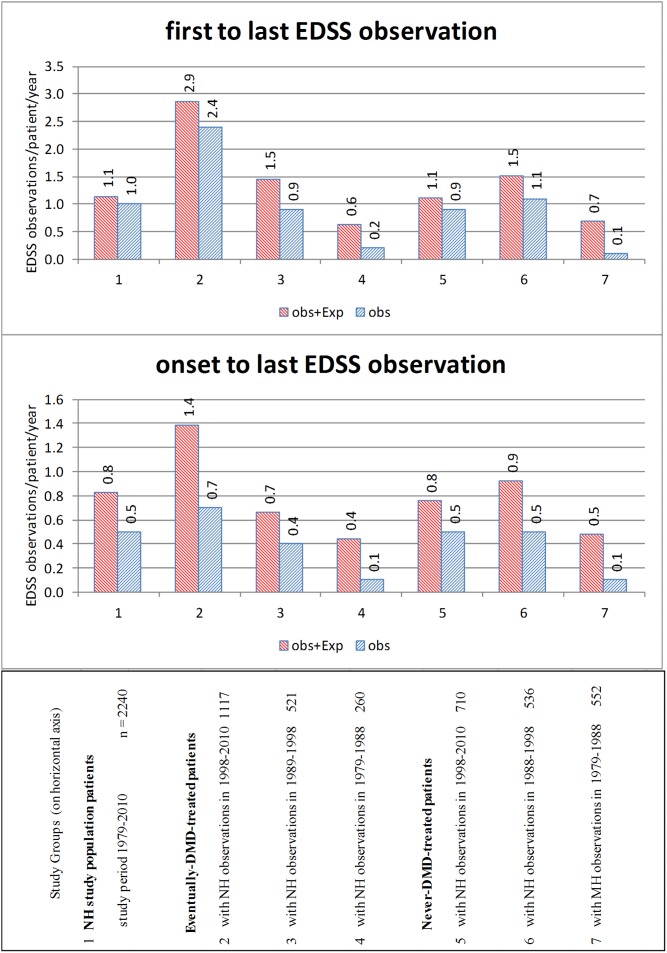
Assessment rates for EDSS clinical observations (obs) and expected midpoint observations (Exp), by relapsing-onset MS study groups and study periods, Nova Scotia, 1979–2010. Footnote: obs = an EDSS clinical observation. obs+Exp = obs and expected EDSS measures, assuming midpoint survival time at irreversible disability endpoints.

Figure 5 and Table S3 describe censoring-bias-size, variance and trends in 1979–2010 Dalhousie Multiple Sclerosis Research Unit clinical observations, as measured by assessment rates. EDSS assessment rates per-patient-per-year from first to last clinical observation and from onset to last clinical observation, by study groups and study periods, are measured. Assessment rates which count both clinical observations (obs) and midpoint survival measures (Exp), as measured between a patient’s first and last clinical observations, are four times greater (2.9/0.7) in 1979–2010 clinical observations from 1,117 eventually-DMD-treated patients (subgroup2) than in 1979–1988 clinical observations from 552 never-DMD-treated patients (subgroup 7); the ratio, as measured between onset and last clinic-visit, is 3∶1 (1.4/0.5).

Progression speed estimates from clinical observations vary inversely with censoring-bias-size, independent of whether maximum, minimum or midpoint survival time is assumed. Censoring-bias-size in Nova Scotia DMSRU clinical observations trended downward from 1979 to 2010. For patients never-treated with disease-modifying-drugs, censoring-bias, as measured by mean years from onset to first clinic-visit observation, fell from 4.4 years in 1979–1998 observations (subgroup 7) to 1.9 years in 1979–2010 observations (subgroup 5); for patients eventually-treated with disease-modifying-drugs, this measure decreased from 3.8 years in 1979–1988 observations (subgroup 4) to 1.1 years in 1979–2010 observations (subgroup 2). For patients never-treated with disease-modifying-drugs, the EDSS assessment rate per-patient-per-year, as measured from onset to last observation, increased from 0.1 in 1979–1988 observations (subgroup 7) to 0.5 in 1998–2010 observations (subgroup 5); comparable assessment rates for patients eventually-treated with disease-modifying-drugs increased from 0.1 (subgroup 4) to 0.7 (subgroup 2).

## Conclusions and Discussion

Estimates of disability progression speed from clinical observations which assume midpoint survival time are significantly faster than estimates which assume maximum survival time. These results suggest that, at least for observations collected at Dalhousie Multiple Sclerosis Research Unit (DMSRU) clinics from 1979–2010, the missing (censored) information between clinical observations is too large and variable to ignore.

This is understandable, in retrospect. The DMSRU evolved from a small solo neurological practice in 1979 to a large university-hospital-based group practice which, in August 1998, became solely responsible for delivering Nova Scotia’s diseases-modifying-drug treatment program for relapsing-onset multiple sclerosis patients. By 2010 the DMSRU served over 90% of Nova Scotia multiple sclerosis patients. Clinical assessments per patient per year were initially infrequent, increased gradually over time, varied greatly within and across study groups, and increased abruptly if patients began disease-modifying-drug treatment. It is therefore not surprising that estimates of progression speed from unadjusted clinical observations, which implicitly assume maximum survival time, vary greatly across study groups and study periods, or that censoring-bias-adjusted estimates, which assume midpoint survival time, are faster and more stable.

Assuming midpoint survival time for events of interest (and creating expected midpoint measures) is a long-established censoring-bias-adjustment method. Assuming midpoint survival time, on average, is defensible when actual times of events of interest are not known. Like other sampling-bias-adjustment methods, censoring-bias-adjustment methods increase the comparability of estimates from observations collected intermittently and at irregular intervals. Unlike other sampling-bias-adjustment methods, censoring-bias-adjusted estimates that assume midpoint survival time may claim, on probabilistic grounds, to be estimates of typical disease progression speed. At the very least, they may claim to be more typical than estimates which assume maximum survival time. While accepting clinical observations at face value is both computationally convenient and defensible when analyzing experimental data collected at frequent and fixed intervals over short periods, such as clinical trials data, this practice may not be defensible when analyzing observations collected at infrequent and irregular intervals over many years [47].

Estimates of typical progression speed are important for stakeholders – such as patients, families, clinicians, epidemiologists, economists, decision-makers, and policy-makers – and they should be aware that estimates from unadjusted clinical observations underestimate typical progression speed to some extent.

What are the implications of our Nova Scotia results for the comparability of multiple sclerosis clinical observations within and across clinics and jurisdictions worldwide? The application of censoring-bias-adjustment methods which assume midpoint survival time to 1979–2010 Nova Scotia clinical observations increased estimates of progression speed and reduced variability in estimates within and across study groups and study periods. Similar results may occur if censoring-bias reduction methods are applied to multiple sclerosis clinical observations worldwide. The only way to find out is to apply these methods to other clinical datasets. Our SAS code is available on request.

What are the implications for effectiveness studies of treatments expected to slow disease progression? To minimize bias in treatment effect estimates that is attributable to censoring-bias, the censoring-bias in treatment-group observations should be as similar as possible to that in natural history comparator-group observations. Treatment effect estimates will be downward-biased, to some degree, whenever censoring-bias in comparator-group observations is greater than in treatment-group observations. This is usually the case in 1979–2010 Nova Scotia observations. If this downward bias is sufficiently large, estimates of treatment effects will be negative, even though the treatment effect is in fact positive. The difference in censoring-bias between treatment-group and comparator-group observations may be reduced by applying a censoring-bias-adjustment method that assumes midpoint survival time. Preliminary investigations, after applying midpoint methods to 1979–2010 Nova Scotia clinical observations, find that censoring-bias-adjusted estimates of treatment effect size tend to be larger than unadjusted estimates, and are more stable within and across study groups and time periods. (We plan to report these results in a paper-in-progress.) Estimates of treatment effect size from censoring-bias-adjusted clinical observations may also claim to be estimates of typical treatment effect size, being derived from estimates of typical disability progression speed before and after treatment.

### Study strengths

The Dalhousie Multiple Sclerosis Research Unit’s systematic assessment of disability at each clinic-visit from 1979–2010 is a strength. Inter-observer measurement variability may be comparatively small from 1979–1998, since most assessments were made by three neurologists (JM, VB, CM). Canada’s federal/provincial universal-coverage hospital and Medicare insurance programs reduced financial barriers to necessary care throughout study period 1979–2010. The Dalhousie Multiple Sclerosis Research Unit evolved from a small solo referral practice in 1979 to a multi-disciplinary university-based practice whose patients, by 2010, were representative of all Nova Scotia’s multiple sclerosis patients [48]. The complex patterns of censoring-bias in 1979–2010 clinical observations serves to illustrate how censoring-bias confounds estimates of typical disability progression speed. It also serves to illustrate the consequences of applying censoring-bias-adjustment methods across study groups and study periods. Although censoring-bias-adjustment methods are not new, to our knowledge this is the first study to apply them to multiple sclerosis clinical observations.

### Overview

This study demonstrates that estimates of typical disability progression speed in definite relapsing-onset multiple sclerosis from clinical observations are sensitive to censoring-bias size and variance, and to analysts’ assumptions about survival time at irreversible disability endpoints. It also demonstrates that estimates of progression speed that assume midpoint survival time are faster, and less variable across study groups and time periods, than estimates that assume maximum survival time. While these results are specific to Nova Scotia 1979–2010 multiple sclerosis clinical observations, they have broader implications. When the missing information between clinical observations is too large and variable to ignore, estimates of typical disease progression from censoring-bias-adjusted observations are more relevant to stakeholders than estimates of atypically slow progression speed from unadjusted observations.

## Supporting Information

Figure S1 **EDSS observations and irreversible progression paths, assuming midpoint survival time, for multiple sclerosis patients ID 1011 and ID 1013 who attended Dalhousie Multiple Sclerosis Research Unit (DMSRU) clinics, Nova Scotia, in period 1979–2010. Footnote:** Estimated irreversible disability (EDSS) progression paths, assuming midpoint survival time, are shown by the line which connects the expected midpoint survival time measures derived from a patient’s incomplete clinical observations. The meaning of 0 thru 5 remains constant even though the symbols (flags) vary across patients. The scale of the plots also varies across patients. 0 = Of 1^st^ clinical observation at an irreversible EDSS endpoint. 1 = Ot a transitorily high clinical observation. 2 = Om an intermediate repeat observation at an irreversible endpoint. 3 = Or the last repeat observation at an irreversible endpoint. 4 = syn an expected EDSS midpoint measure. 5 = Oo a synthetic observation at assessed year of MS onset, assuming EDSS = 0.
(TIF)

Figure S2 **EDSS observations and irreversible progression paths, assuming midpoint survival time, for multiple sclerosis patients ID 1051 and ID 1033, who attended Dalhousie Multiple Sclerosis Research Unit (DMSRU) clinics, Nova Scotia, in period 1979–2010. Footnote:** Estimated irreversible disability (EDSS) progression paths, assuming midpoint survival time, are shown by the line which connects the expected midpoint survival time measures derived from a patient’s incomplete clinical observations. The meaning of 0 thru 5 remains constant even though the symbols (flags) vary across patients. The scale of the plots also varies across patients. 0 = Of 1^st^ clinical observation at an irreversible EDSS endpoint. 1 = Ot a transitorily high clinical observation. 2 = Om an intermediate repeat observation at an irreversible endpoint. 3 = Or the last repeat observation at an irreversible endpoint. 4 = syn an expected EDSS midpoint measure. 5 = Oo a synthetic observation at assessed year of MS onset, assuming EDSS = 0.
(TIF)

Table S1 **Expanded Disability Status Scale (EDSS).**
(TIF)

Table S2 **Estimates of disability progression per year in relapsing-onset multiple sclerosis natural history study groups, assuming midpoint or maximum survival time, DMSRU, Nova Scotia, 1979–2010.** Footnote: DMSRU = Dalhousie Multiple Sclerosis Research Unit. DMD = disease-modifying-drugs. βyso = estimated annual EDSS change per year-since-onset, using a fixed effects regression model.
(TIF)

Table S3 **EDSS assessment rates, by study groups and study periods, Nova Scotia, 1979–2010.** Footnote: DMSRU = Dalhousie Multiple Sclerosis Research Unit. DMD = disease-modifying-drugs. (a) obs = an EDSS clinical-observation from 0–9.5; (b) EDSS = 0 at onset, by assumption, and Exp = an expected EDSS measure, assuming midpoint survival time at irreversible disability endpoints; (c) left-censoring = missing observations between assessed year-of-onset and first clinical observation; for patients with Exp measures, years-since-onset to the 1st EDSS observation was computed using their first Exp measure; (d) interval-censoring = missing observations between first and last clinical observation; (e) right-censoring = missing observations after last clinical observation; right-censoring-bias-size in Kaplan Meier synthetic onset cohorts, which increases as the chosen analytical time horizon increases, is not reported.
(TIF)
