# Supplementary figures and images for: Estimating Typical Multiple Sclerosis Disability Progression Speed from Clinical Observations
Source: PLoS One. 2014 Oct 17;9(10):e105123. doi: 10.1371/journal.pone.0105123 (PMC4201451; doi:10.1371/journal.pone.0105123)

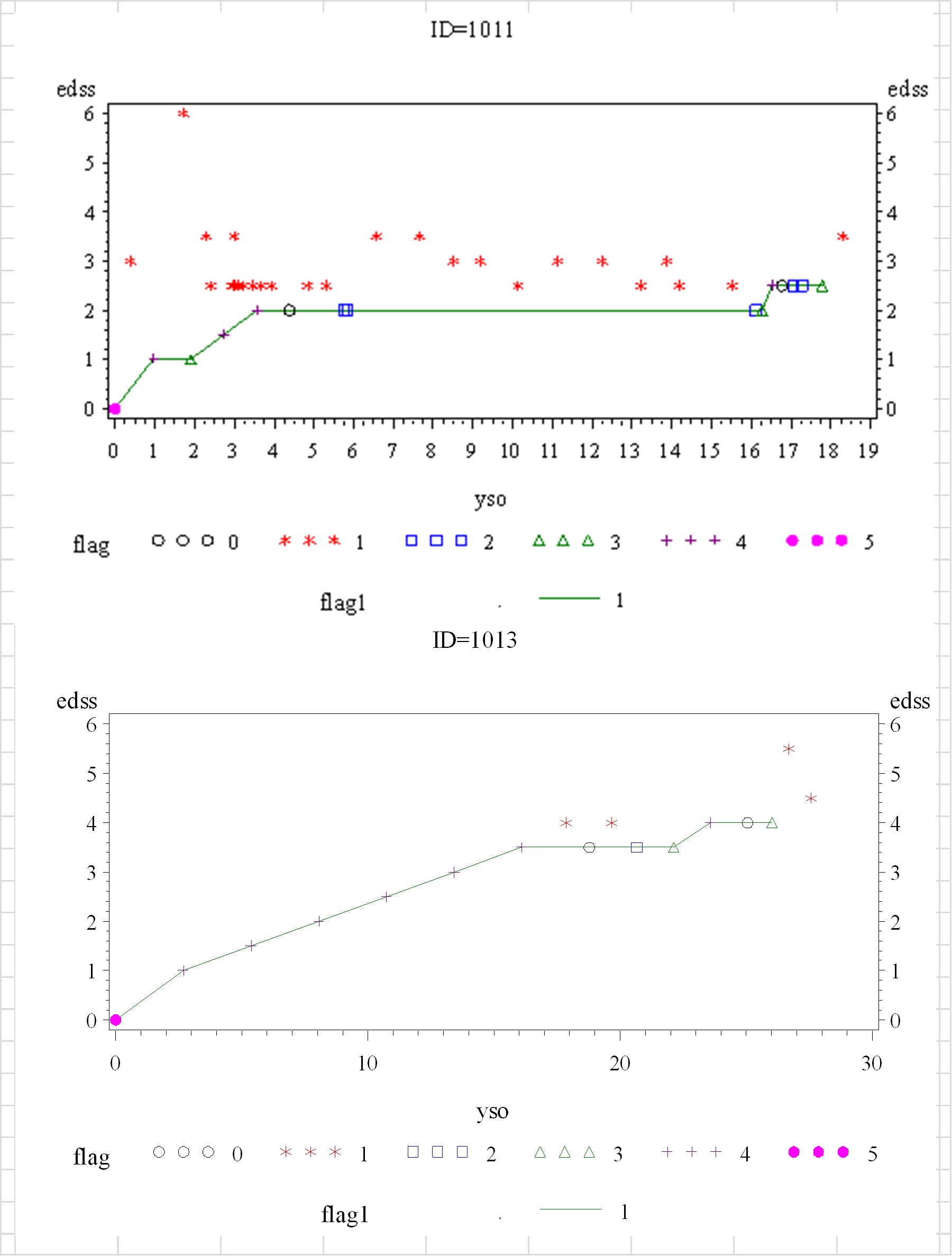

Supplement: Figure S1 — EDSS observations and irreversible progression paths, assuming midpoint survival time, for multiple sclerosis patients ID 1011 and ID 1013 who attended Dalhousie Multiple Sclerosis Research Unit (DMSRU) clinics, Nova Scotia, in period 1979–2010. Footnote: Estimated irreversible disability (EDSS) progression paths, assuming midpoint survival time, are shown by the line which connects the expected midpoint survival time measures derived from a patient’s incomplete clinical observations. The meaning of 0 thru 5 remains constant even though the symbols (flags) vary across patients. The scale of the plots also varies across patients. 0 = Of 1st clinical observation at an irreversible EDSS endpoint. 1 = Ot a transitorily high clinical observation. 2 = Om an intermediate repeat observation at an irreversible endpoint. 3 = Or the last repeat observation at an irreversible endpoint. 4 = syn an expected EDSS midpoint measure. 5 = Oo a synthetic observation at assessed year of MS onset, assuming EDSS = 0. (TIF) [file pone.0105123.s001.tif]

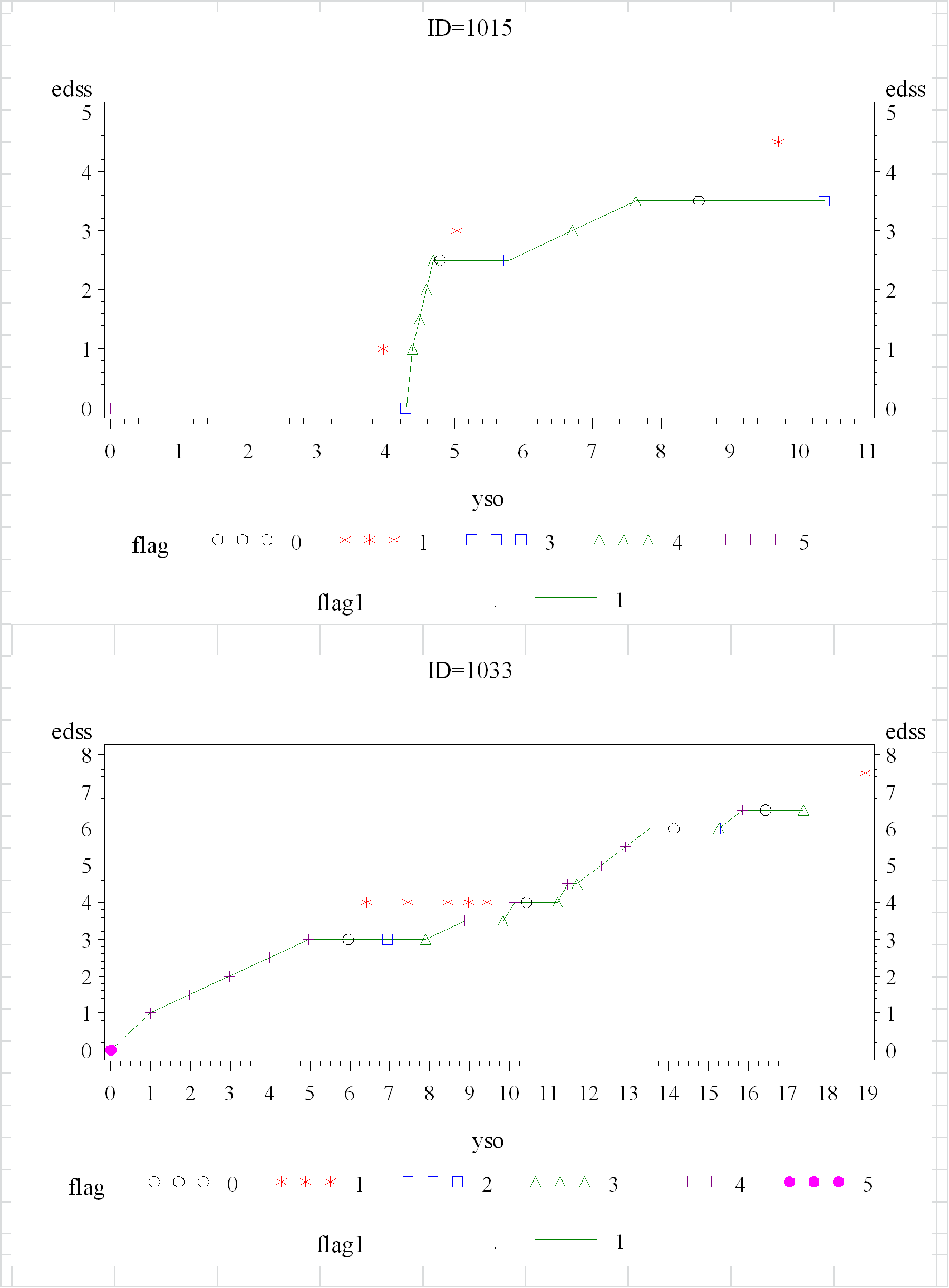

Supplement: Figure S2 — EDSS observations and irreversible progression paths, assuming midpoint survival time, for multiple sclerosis patients ID 1051 and ID 1033, who attended Dalhousie Multiple Sclerosis Research Unit (DMSRU) clinics, Nova Scotia, in period 1979–2010. Footnote: Estimated irreversible disability (EDSS) progression paths, assuming midpoint survival time, are shown by the line which connects the expected midpoint survival time measures derived from a patient’s incomplete clinical observations. The meaning of 0 thru 5 remains constant even though the symbols (flags) vary across patients. The scale of the plots also varies across patients. 0 = Of 1st clinical observation at an irreversible EDSS endpoint. 1 = Ot a transitorily high clinical observation. 2 = Om an intermediate repeat observation at an irreversible endpoint. 3 = Or the last repeat observation at an irreversible endpoint. 4 = syn an expected EDSS midpoint measure. 5 = Oo a synthetic observation at assessed year of MS onset, assuming EDSS = 0. (TIF) [file pone.0105123.s002.tif]

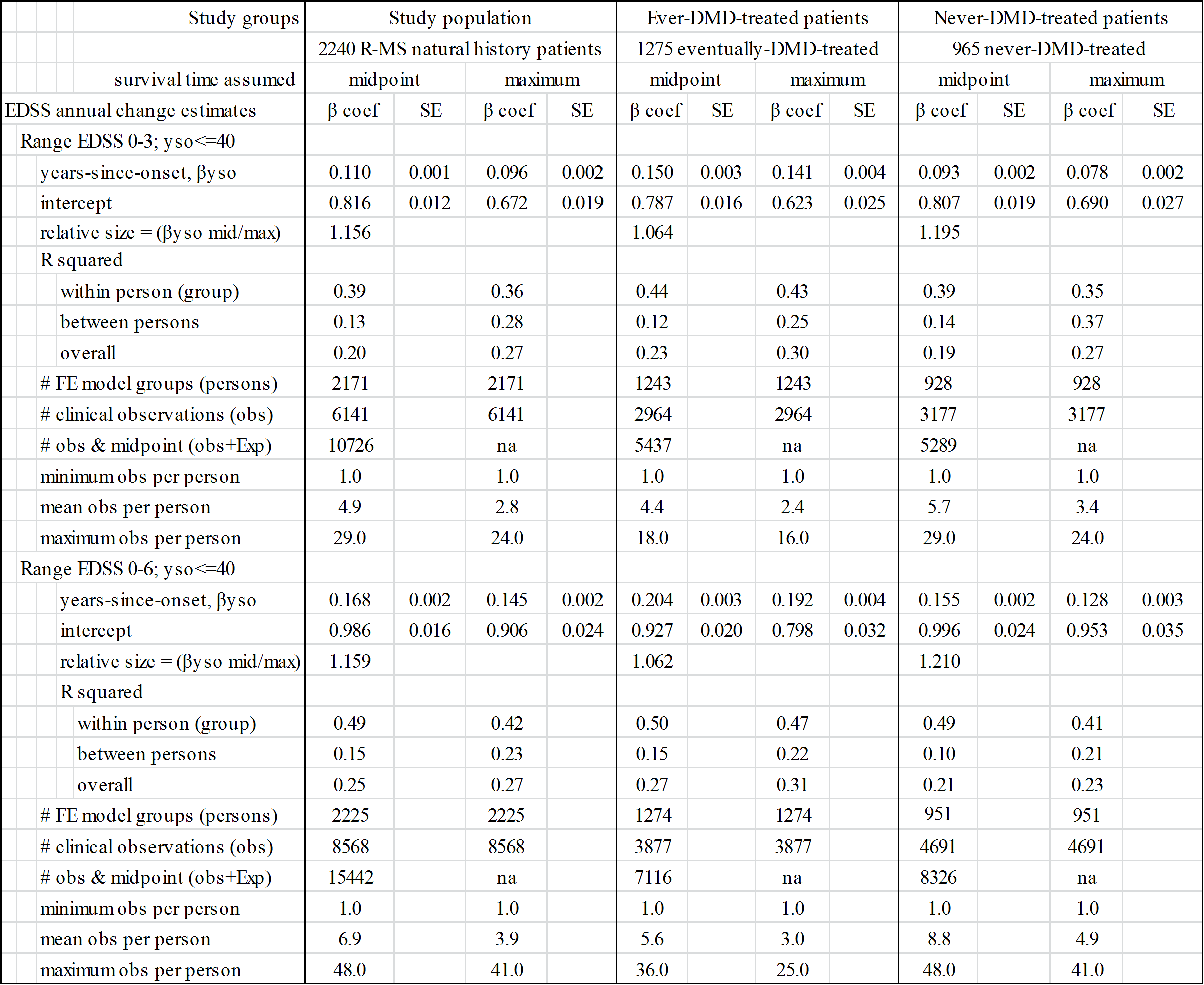

Supplement: Table S2 — Estimates of disability progression per year in relapsing-onset multiple sclerosis natural history study groups, assuming midpoint or maximum survival time, DMSRU, Nova Scotia, 1979–2010. Footnote: DMSRU = Dalhousie Multiple Sclerosis Research Unit. DMD = disease-modifying-drugs. βyso = estimated annual EDSS change per year-since-onset, using a fixed effects regression model. (TIF) [file pone.0105123.s004.tif]
